# NOS2-deficient mice with hypoxic necrotizing lung lesions predict outcomes of tuberculosis chemotherapy in humans

**DOI:** 10.1038/s41598-017-09177-2

**Published:** 2017-08-18

**Authors:** Martin Gengenbacher, Maria A. Duque-Correa, Peggy Kaiser, Stefanie Schuerer, Doris Lazar, Ulrike Zedler, Stephen T. Reece, Amit Nayyar, Stewart T. Cole, Vadim Makarov, Clifton E. Barry III, Véronique Dartois, Stefan H. E. Kaufmann

**Affiliations:** 1 Max Planck Institute for Infection Biology, Department of Immunology, Berlin, Germany; 2 Public Health Research Institute, Rutgers, The State University of New Jersey, Newark, NJ USA; 3 Tuberculosis Research Section, Laboratory of Clinical Infectious Diseases, National Institute of Health-National Institute of Allergy and Infectious Diseases, Bethesda, MD USA; 4 Global Health Institute, École Polytechnique Fédérale de Lausanne, Lausanne, Switzerland; 5 A. N. Bakh Institute of Biochemistry, Russian Academy of Science, Moscow, Russia; 6 Institute of Infectious Disease and Molecular Medicine, Faculty of Health Sciences, University of Cape Town, Rondebosch, Republic of South Africa; 7 Wellcome Trust Sanger Institute, Cambridge, United Kingdom; 8 University of Cambridge School of Clinical Medicine, Cambridge, United Kingdom; 9 Albany Molecular Research Inc, Singapore, Singapore

## Abstract

During active TB in humans a spectrum of pulmonary granulomas with central necrosis and hypoxia exists. BALB/c mice, predominantly used in TB drug development, do not reproduce this complex pathology thereby inaccurately predicting clinical outcome. We found that *Nos2* ^−/−^ mice incapable of NO-production in immune cells as microbial defence uniformly develop hypoxic necrotizing lung lesions, widely observed in human TB. To study the impact of hypoxic necrosis on the efficacy of antimycobacterials and drug candidates, we subjected *Nos2* ^−/−^ mice with TB to monotherapy before or after establishment of human-like pathology. Isoniazid induced a drug-tolerant persister population only when necrotic lesions were present. Rifapentine was more potent than rifampin prior to development of human-like pathology and equally potent thereafter, in agreement with recent clinical trials. Pretomanid, delamanid and the pre-clinical candidate BTZ043 were bactericidal independent of pulmonary pathology. Linezolid was bacteriostatic in TB-infected *Nos2* ^−/−^ mice but significantly improved lung pathology. Hypoxic necrotizing lesions rendered moxifloxacin less active. In conclusion, *Nos2* ^−/−^ mice are a predictive TB drug development tool owing to their consistent development of human-like pathology.

## Introduction

Tuberculosis (TB) is a global health threat further worsened by increasing incidences of drug resistance and HIV co-infection^1^. New drugs to combat resistant TB and to shorten the current 6 to 9 month standard-of-care chemotherapy are urgently needed. In most cases, infection starts in the lungs where *Mycobacterium tuberculosis* induces well-structured granulomas composed of various hematopoietic cells in which the pathogen is contained^2, 3^. During progression to active disease, infected macrophages and neutrophils undergo necrosis at the core of these structure, resulting in the formation of a hypoxic caseum^2^.^18^F-fluorodeoxyglucose positron emission tomography/computed tomography has revealed that pulmonary TB is a highly dynamic disease: coexisting inflammatory hotspots in lungs of TB patients increase in size, shrink or even resolve over time while others are newly formed^4, 5^. Recent studies have indicated that pro- and anti-inflammatory signals are spatially segregated within granulomas of humans suggesting that granuloma formation in TB is a direct consequence of the local activity of inflammatory pathways^6^.

Among the available animal models, non-human primates mirror the facets of human TB best, but broad application is hampered by prohibitive cost and ethical concerns. To date, mice remain the most widely used model for pre-clinical TB drug development. However, upon *M. tuberculosis* infection, small rodents develop a more uniform type of unstructured lesions that lack central necrosis and hypoxia^7^, two key features of human granulomas. Commonly employed strains such as BALB/c mice may therefore inaccurately predict the outcome of new drug candidates and treatment regimens in humans, as emphasized by the disappointing results of three large and costly clinical trials^8–10^. Bridging the disconnect between pre-clinical mouse models and TB patients is therefore mandatory for accelerated drug development.

The murine supersusceptibility to tuberculosis 1 (*sst1*) locus has been found to prevent formation of necrotic lesions in mice. The intracellular pathogen resistance 1 (Ipr1)-protein encoded within this locus can switch the molecular program of infected macrophages from necrosis to apoptosis^11^. C3HeB/FeJ mice lacking a functional *ipr1* gene develop large pulmonary lung lesions with central caseous necrosis during *M. tuberculosis* infection^12^. This model has been used in several studies to evaluate monotherapy with anti-TB drugs and clinically relevant drug regimens^12–17^. In-depth pathological characterization of *M. tuberculosis*-infected C3HeB/FeJ mice revealed multiple lung lesion types which are either large, caseous and necrotic and characterized by neutrophil-dominated granulocytic pneumonia or which reflect unstructured lesions as observed in BALB/c mice^18^. C3HeB/FeJ mice with a predominance of caseous necrotic lesions versus cellular inflammatory lesions (‘BALB/c-type’) respond differently to chemotherapy^14^.

TB drugs need to penetrate the granuloma to act on the pathogen in different physiological states. Previous *in vitro* studies suggest that drug penetration into non-replicating *M. tuberculosis* is reduced^19^. Such non-replicating persisters are a difficult-to-treat population believed to reside in the unfavourable microenvironment of necrotic and hypoxic granulomas^2^. Moreover, drugs have been shown to differentially penetrate into well-structured TB lesions in rabbits^20^ and in humans^21^. Moxifloxacin (MXF) in particular was demonstrated to diffuse relatively poorly into central caseum – a finding which can explain the failure of this drug to shorten treatment duration in the clinic^9, 10, 21^. Thus, a substantial body of evidence suggests that different granuloma pathologies as they occur in TB patients impact treatment outcomes.

The inducible oxygen-dependent host enzyme nitric oxide synthase-2 (NOS2) counteracts microbial intruders by means of NO production. Consequently, *Nos2* ^−/−^ mice deficient for this defence mechanism are exquisitely susceptible to bacterial infections including TB^22^. Our previous work demonstrated that *M. tuberculosis*-infected *Nos2* ^−/−^ mice develop solid structured granulomas in which central necrosis followed by hypoxia can be induced by neutralization of interferon-gamma or tumour necrosis factor alpha (TNF-α)^23, 24^. The human-like granuloma pathology of *Nos2* ^−/−^ mice prompted us to harness this model for evaluation of TB monotherapy with first-line drugs and new candidates under development, to examine the direct impact of necrosis and hypoxia on outcome of chemotherapy.

## Results

### Dermal *M. tuberculosis* infection of *Nos2*^−/−^ mice consistently induces lung lesions with central necrosis and hypoxia

**Figure 1:**
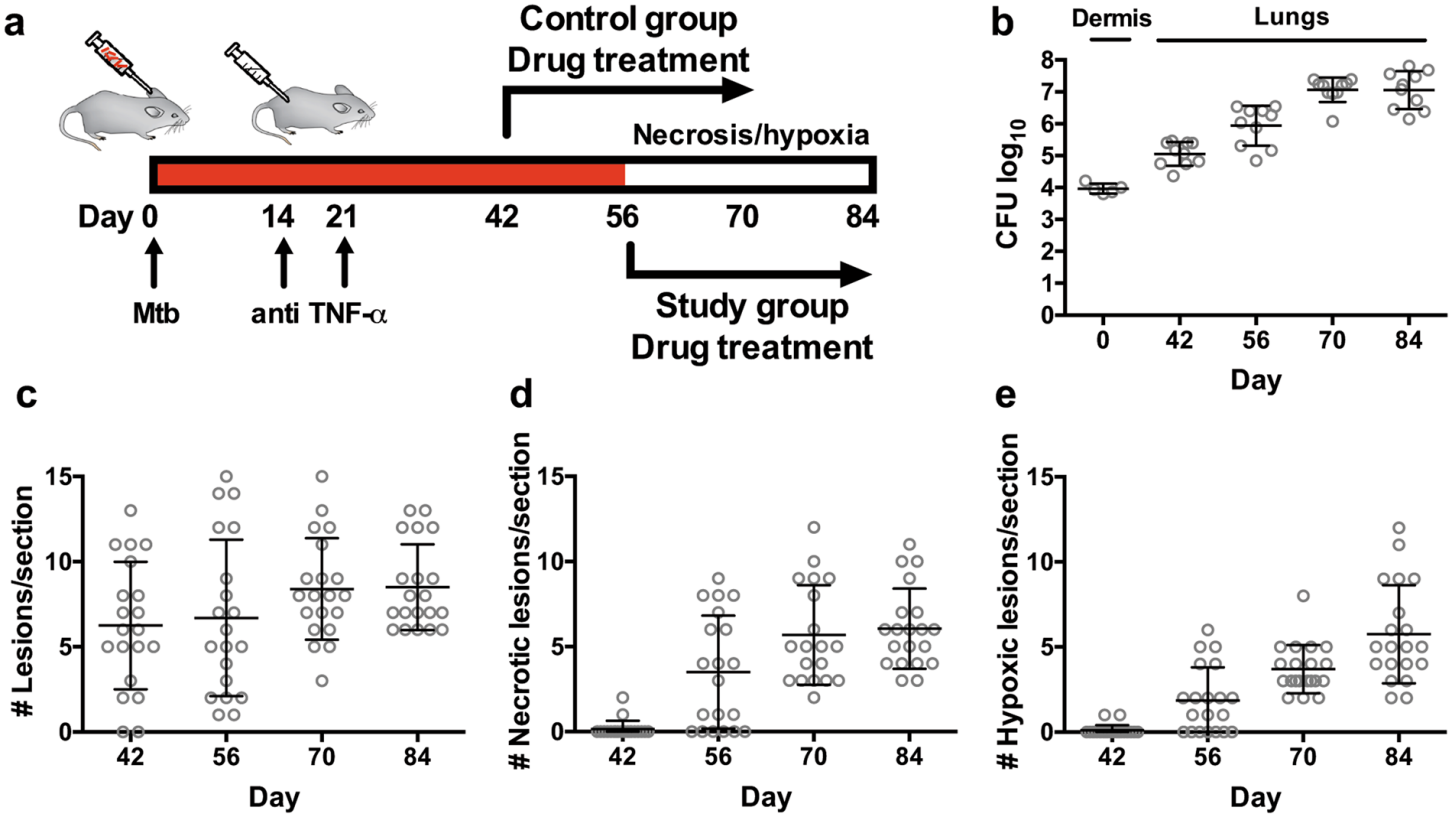
Temporal development of hypoxic necrotizing lung lesions in *Nos2* ^−/−^ mice upon dermal *M. tuberculosis* infection. (**a**) Schematic representation of study design. (**b**) Infection dose (day 0) and bacterial burden in lungs at 42, 56, 70 and 84 days post-intradermal *M. tuberculosis* infection determined by plating serial dilutions of lung homogenates on agar. n = 10, representative of 2 independent experiments. (**c**–**e**) Histopathological evaluation of sections obtained from the entire left lung lobe of *M. tuberculosis*-infected *Nos2* ^−/−^ mice at designated time points. Consecutive sections were stained with H&E to quantify lesions (**c**) and necrosis (**d**). Hypoxic lesions were identified by detection of pimonidazole adducts (Supplementary Fig. S1e), n = 5, representative of 3 independent experiments. Data are represented as means ± s.d. TNF-α, tumour necrosis factor alpha.

Previously, we demonstrated the capacity of *Nos2* ^−/−^ mice to develop a TB patient-like pulmonary pathology upon dermal *M. tuberculosis* infection that includes central necrosis and hypoxia in structured lesions^23, 24^. In order to harness *Nos*2^−/−^ mice for TB drug development as schematically depicted in Fig. 1a, the temporal onset of patient-like pathology needed to be defined. We dermally infected groups of mice, neutralized the TNF-α response at 14 and 21 days post-infection, and assessed bacterial burden (superior, middle, inferior and post-caval lobe) and pathology (left lobe) of lungs at days 42, 56, 70 and 84. The total number of bacilli residing in lungs of *Nos*2^−/−^ mice was 10^5^ colony forming units (c.f.u.) at day 42 and increased to 10^7^ c.f.u. by day 70 after which the bacterial burden remained stable on a level comparable to low-dose-infected BALB/c mice (Fig. 1b)^25^. Consecutive thin sections of the fixed and paraffin-embedded left lung lobe were stained with H&E or used for immunohistochemistry to visualize hypoxic areas. The average number of lesions per section moderately increased from 6.3 at day 42 to 8.5 at the end of the experiment (Fig. 1c). In contrast, the numbers of necrotic and hypoxic lesions were negligible 42 days post-infection, became apparent at day 56 and further increased until 84 days, when roughly 75% of all lesions showed patient-like pathology including necrosis and hypoxia (Fig. 1d,e). Importantly, lung lesions of all mice collectively and co-ordinately developed advanced pathology in a distinctively narrow time window. We concluded that the response of hypoxic necrotizing pulmonary lesions to TB chemotherapy in *Nos*2^−/−^ mice could be assessed by initiating drug treatment at day 42 in the control group and after onset of necrosis and hypoxia at day 56 in the study group (Fig. 1a).

### Pulmonary pathology of *M. tuberculosis*-infected *Nos2*^−/−^ mice includes central necrosis, hypoxia, foamy macrophages and peripheral fibrosis

With few exceptions, lung lesions 42 days after infection appeared non-necrotizing and not hypoxic (Supplementary Fig. S1a). At day 56, about 50% of all lesions showed signs of central necrosis of which half were hypoxic (Supplementary Fig. S1a,b). Pimonidazole adducts formed under oxygen limitation often appeared in regions with circular shape. (Supplementary Fig. S1a, lower row, Supplementary Fig. S1b, middle panel). Acid-fast staining of lung sections revealed a large number of *M. tuberculosis* bacilli in lesion centres at onset of necrosis (Supplementary Fig. S1b, right panel). From day 70 onwards, lighter stained central areas of lung lesions were enlarged and sharply separated from the surrounding granulomatous inflammatory infiltrates indicating advanced necrosis (Supplementary Fig. S1a, upper panel).

**Figure 2:**
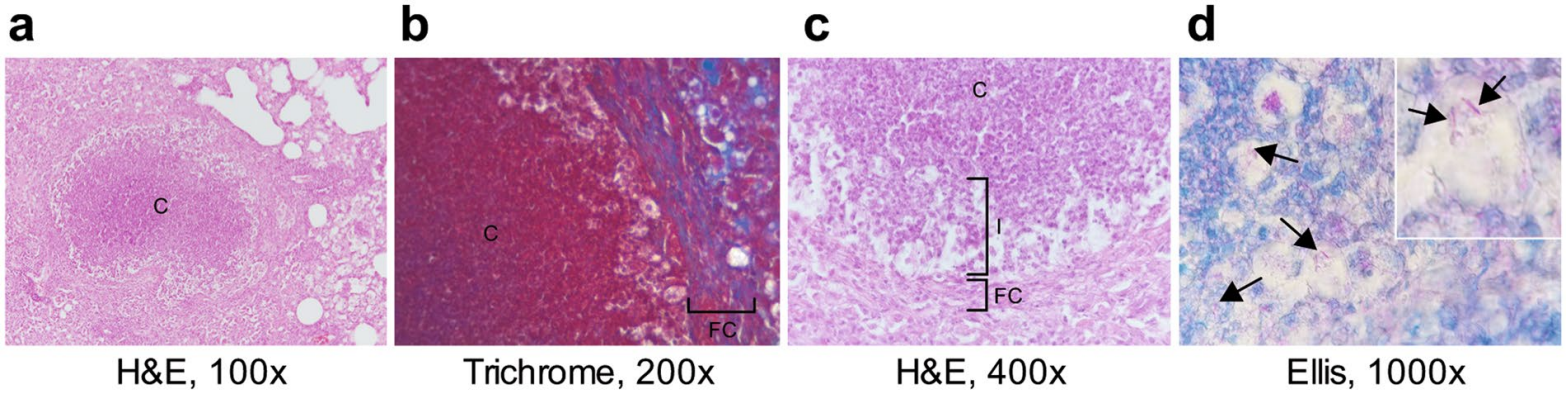
*Nos2* ^−/−^ mice with TB form pulmonary caseous lesions with fibrous encapsulation and infected cells. (**a**–**d**) Lung sections of *Nos2* ^−/−^ mice 84 days post-intradermal *M. tuberculosis* infection. H&E, Trichrome and Ellis’ acid fast staining of caseous lesions are shown. (**a**) The caseous center is separated from the fibrous cuff consisting of blue-stained newly formed collagen fibers (**b**) by a layer of lighter-appearing cells (**c**) presumably including infected foamy macrophages. (**d**) Acid fast rod-shaped *M. tuberculosis* bacilli are stained pink (arrows). Abbreviations: C, caseation; I, intersection; FC, fibrous cuff; H&E, haematoxylin & eosin.

On day 84 we observed advanced necrosis characterized by enlarged areas of central tissue consolidation, fibrous encapsulation and caseation of lesions on day 84, which are pathological late-stage correlates of human TB (Fig. 2a,b). The fibrous capsule was separated from the central caseum by a layer of infected cells presumably foamy macrophages (Fig. 2c,d) as seen in human granulomas^26^. In contrast to other murine TB models, lung lesions of *Nos*2^−/−^ mice collectively and coordinately developed hypoxic necrotizing lung lesions allowing to assess the direct impact of TB chemotherapy on patient-like pathology by initiating drug treatment at different time points (Fig. 1a).

### Efficacy of isoniazid monotherapy is reduced in hypoxic necrotizing lesions

**Figure 3:**
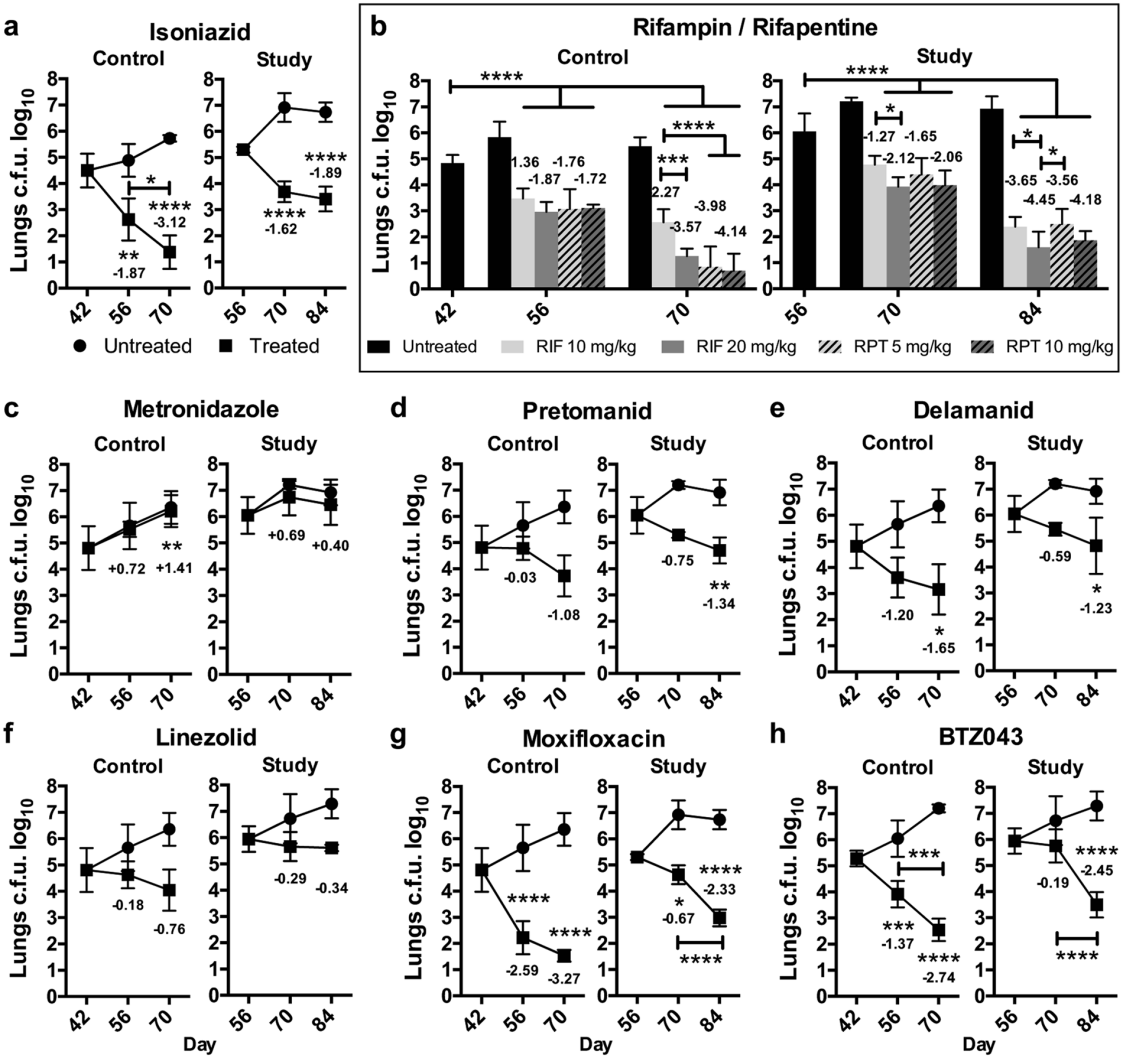
Impact of monotherapy on pulmonary bacterial burden in *Nos2* ^−/−^ mice with TB. Monotherapy for 4 weeks was initiated at day 42 (control group) or after onset of central necrosis and hypoxia in lung lesions at day 56 (study group) as described in Fig. 1a. Drugs were administered by oral gavage on 6 days per week. Bacterial burdens in lungs were assessed by plating organ homogenates on agar. One representative dataset out of 2 independent experiments is shown. Data were analysed using two-way ANOVA with multicomparison and Tukey’s post-test. Shown are means ± s.e.m. and the log10 reduction of mean c.f.u. counts as compared to animals prior to chemotherapy (summarized in Supplementary Table S1), n = 5, *p < 0.05, **p < 0.01, ***p < 0.001, ****p < 0.0001. (**a**) isoniazid, 25 mg/kg. (**b**) rifampin, 10 mg/kg and 20 mg/kg; rifapentine, 5 mg/kg and 10 mg/kg. (**c**) metronidazole, 400 mg/kg. (**d**) pretomanid, 75 mg/kg. (**e**) delamanid, 1 mg/kg. (**f**) linezolid, 100 mg/kg. (**g**) moxifloxacin, 200 mg/kg. (**h**) BTZ043, 50 mg/kg.

The first-line TB drug isoniazid (INH) targets mycobacterial cell wall biosynthesis and kills growing *M. tuberculosis* but is well tolerated by the non-replicating pathogen *in vitro* ^27, 28^ and *in vivo* ^29^. INH at a dose of 25 mg/kg was strongly bactericidal in *Nos2* ^−/−^ mice when chemotherapy was started 42 days post-infection (Fig. 3a). The initial steep decline in pulmonary bacterial burden slowed down after 14 days of drug treatment revealing a bi-phasic profile comparable to observations made in BALB/c mice (Fig. 3a)^13^. In contrast, when INH administration in *Nos2* ^−/−^ mice was initiated after onset of necrosis and hypoxia the bacterial load declined by 1.62 log c.f.u. during the first 2 weeks of treatment and remained an a comparable level thereafter (Fig. 3a). Development of genetic drug resistance that might have prevented further decline in c.f.u. could be ruled out since plating lung homogenates on agar containing 4 μg/ml INH at the end of the experiment did not result in colony formation. We conclude that a drug-tolerant persister population of *M. tuberculosis* developed in hypoxic necrotizing lung lesions of *Nos2* ^−/−^ mice under INH pressure.

### Comparable bactericidal activity of rifampin and rifapentine

Rifamycins inhibit RNA biosynthesis and are a cornerstone of current TB chemotherapy^30^. While rifampin (RIF) has been in clinical use for decades, its derivative rifapentine (RPT) showed higher potency and greater drug exposure in mice, thereby raising hopes for shorter treatment over RIF^31–33^. Both drugs, RIF and RPT were profoundly bactericidal in *Nos2* ^−/−^ mice (Fig. 3b). We observed a significant dose response for RIF administered at 10 or 20 mg/kg, which was only moderately influenced by the presence of hypoxic necrotizing lesions at treatment start. In contrast, RPT did not show a dose response when given at 5 and 10 mg/kg (Fig. 3b). The bacterial load of lungs in groups receiving 20 mg/kg RIF, 5 mg/kg RPT and 10 mg/kg RPT were roughly equivalent in absence of necrosis and hypoxia (Fig. 3b, left panel). RIF was more active in patient-like lesions in *Nos2* ^−/−^ mice while RPT efficacy did not change with pathology: the burden of pulmonary TB at the end of the experiment was comparable in mice treated with 10 mg/kg RIF and 10 mg/kg RPT (Fig. 3b, right panel) principally resembling clinical outcome^8^. Organ homogenates plated on agar containing either RIF or RPT did not result in any c.f.u., confirming absence of genetic drug resistance. Taken together, RIF and RPT had similar efficacy in *Nos2* ^−/−^ mice with hypoxic necrotizing lung lesions.

### Pretomanid and delamanid but not metronidazole are bactericidal in hypoxic necrotizing lesions

The antibiotic metronidazole (MTZ) can kill non-replicating *M. tuberculosis* under hypoxic *in vitro* conditions, though only at very high concentrations that are hardly achieved *in vivo* ^34^. Daily administration of 400 mg/kg MTZ to *Nos2* ^−/−^ mice had no effect on c.f.u. recovered from lung homogenates (Fig. 3c). A minute reduction after onset of necrosis and hypoxia in lung lesions (Fig. 3c) may be taken as indication for a hypoxia induced non-replicating *M. tuberculosis* subpopulation. In contrast, two chemically related derivatives, pretomanid (PTM) and delamanid (DLM), were bactericidal in *Nos2* ^−/−^ mice with or without hypoxic necrotizing lesions (Fig. 3d,e). PTM is in advanced clinical development and DLM has recently been approved for use in patients with multidrug-resistant TB. Both drugs are bactericidal for replicating and non-replicating *M. tuberculosis* in culture and in mice that do not develop hypoxic necrotizing TB lesions^35, 36^. Our data support further development of PTN and DLM for TB chemotherapy.

### Hypoxic necrotizing lesions render linezolid bacteriostatic, reduce activity of moxifloxacin and do not affect efficacy of BTZ043

The antimicrobial linezolid (LZD) is in clinical use for treatment of serious infections caused by Gram-positive pathogens including vancomycin-resistant enterococci, methicillin-resistant *Staphylococcus aureus* and penicillin-resistant *Streptococcus pneumoniae* ^37^. The drug is bactericidal in the BALB/c mouse model of TB, in C3HeB/FeJ mice with patient-like pathology and in patients with extensively drug-resistant TB^13, 38^. At a daily dose of 100 mg/kg given to *Nos2* ^−/−^ mice for 4 weeks, LZD caused only marginal statistically insignificant reduction of lung c.f.u., as compared to treatment start (Fig. 3f). The presence of hypoxic necrotizing lesions abolished this trend suggesting bacteriostatic activity of LZD in this model (Fig. 3f).

The fourth generation fluoroquinolone, moxifloxacin (MXF), is broadly used for treatment of bronchitis, pneumonia and bacterial infections of the sinus, skin and abdomen. MXF has a high potency against *M. tuberculosis* in cultures and in mice without human-like lung pathology^39^. In our experiments, onset of hypoxia and necrosis in lung lesions of *Nos2* ^−/−^ mice caused a 10-fold reduction of MXF bactericidal activity (Fig. 3g), indicating a direct impact of human-like pathology on MXF activity. The benzothiazinone pre-clinical drug candidate BTZ043, targeting *M. tuberculosis* cell wall biosynthesis, has nanomolar whole-cell activity and good *in vivo* efficacy in mice^40^. Unlike MFX, BTZ043 was highly potent in *Nos2* ^−/−^ mice regardless of patient-like lung pathology (Fig. 3h).

### Chemotherapy alters TB lung pathology in *Nos2*^−/−^

**Figure 4:**
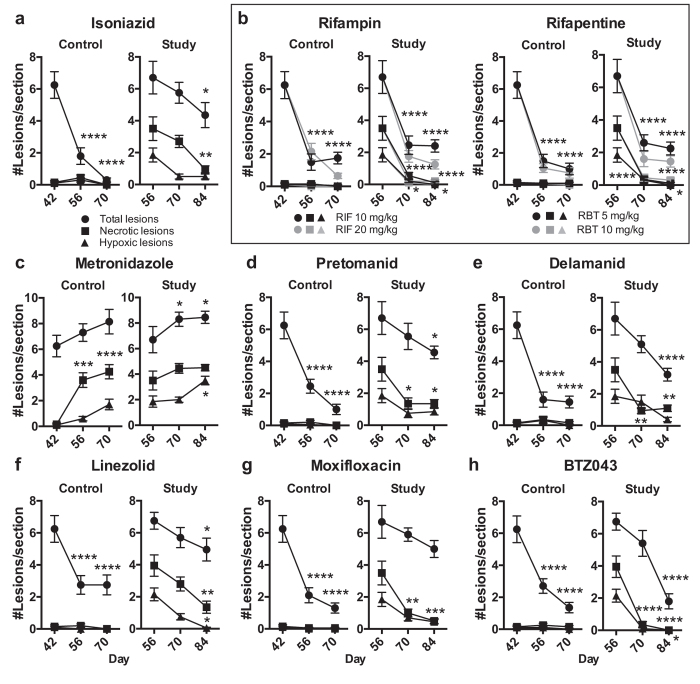
Impact of monotherapy on pulmonary pathology in *Nos2* ^−/−^ mice with TB. Monotherapy for 4 weeks was initiated at day 42 (control group) or after onset of central necrosis and hypoxia in lung lesions at day 56 (study group) as described in Fig. 1a. Drugs were administered by oral gavage on 6 days per week. Consecutive thin sections of the formalin-fixed paraffin-embedded entire left lung lobe were stained with H&E or the pimonidazole hypoxia detection method and analyzed microscopically. One representative dataset out of 2 independent experiments is shown. Data were analyzed using two-way ANOVA with multicomparison and Tukey’s post-test. Shown are means ± s.e.m. and statistical significance as compared to the control group prior to drug treatment, n = 5, *p < 0.05, **p < 0.01, ***p < 0.001, ****p < 0.0001. (**a**) isoniazid, 25 mg/kg. (**b**) rifampin, 10 mg/kg and 20 mg/kg; rifapentine, 5 mg/kg and 10 mg/kg. Statistical significance for different doses of the same drug at respective time points was similar and was only plotted once. (**c**) metronidazole, 400 mg/kg. (**d**) pretomanid, 75 mg/kg. (**e**) delamanid, 1 mg/kg. (**f**) linezolid, 100 mg/kg. (**g**) moxifloxacin, 200 mg/kg. (**h**) BTZ043, 50 mg/kg.

For most drugs the impact of monotherapy on bacterial survival correlated fairly well with histopathological findings (Fig. 4). With the exception of MTZ, none of the study drugs facilitated development of advanced pathology when chemotherapy was initiated prior onset of hypoxia and necrosis (Fig. 4). Unlike RIF and BTZ043, the capacity of INH, RBT, PTM, DLM, LZD and MXF to resolve lung lesions was reduced (Fig. 4). MTZ closely resembled lesion occurrence in untreated animals and thus completely failed to alter pathology (Fig. 4c; Fig. 1c-d). INH, PTM, DLM and MXF maintained a small number of hypoxic necrotizing lesions until end of the experiment at day 84 eventually providing a niche for non-replicating pathogen survival (Fig. 4a,c-d,f). Only RIF and BTZ043 completely abolished hypoxia and necrosis in line with the strong bactericidal activity of both drugs (Fig. 3b,h; Fig. 4b,h). Intriguingly, LZD, bacteriostatic (Fig. 3f) in the presence of human-like lesions, significantly reduced occurrence of hypoxia, necrosis and overall numbers of lesions (Fig. 4f, Supplementary Fig. S2), which leaves the possibility that LZD may unfold more pronounced bactericidal activity after resolving advanced pathology.

## Discussion

The *Nos2* ^−/−^ mouse model of TB evaluated here displays significantly improved lung pathology over conventional BALB/c mice, comprising well-structured lesions with central necrosis and hypoxia as well as peripheral fibrosis (Supplementary Fig S1, Fig. 2). Following dermal infection, inflammatory foci in lungs progressed towards more advanced pathology in all infected mice, showing first signs of central necrosis followed by hypoxia, advanced necrosis and finally caseation in distinct lesions (Fig. 1c-d, Fig. 2). Pimonidazole adducts as indicators of hypoxia frequently appeared annular in the lesional centre indicating advanced necrosis and absence of viable cells capable of metabolizing the hydroxyprobe in the innermost centre. Our model presents similarities with the C3HeB/FeJ mouse model, but has the advantage of consistent and reproducible progression towards necrotic hypoxic lesions in all infected mice. *M. tuberculosis* infection of C3HeB/FeJ mice induces three distinct types of lesions: large patient-like lesions (Type I), rapidly progressive granulocytic lesions mainly composed of neutrophils (Type II) and small cellular inflammatory lesions (Type III, ‘BALB/c-type’)^18^. The heterogeneous distribution of lesion types and their differential responses to chemotherapy, as well as the early mortality of mice predominantly suffering from Type II lesions, remain major challenges of the C3HeB/FeJ mouse model of TB^14^. Type I lesions in C3HeB/FeJ mice were reported to develop liquefaction and even cavitation of necrotic lesions^13^, two pathological end-stage correlates of human TB that were not observed in *Nos2* ^−/−^ mice before termination of our experiments at 84 days after infection (Supplementary Fig. S1). At this late stage of infection, a layer of infected foamy macrophages located on the inner side of the fibrotic capsule constitutes the interface between the cellular and caseous regions as seen in human granulomas^26^ (Fig. 2). Foamy macrophages harbour large quantities of lipids including cholesterol^41^ and presumably undergo necrosis thereby contributing lipids to the caseous centre. Cholesterol is likely one of the major carbon sources of mycobacteria during infection as suggested by transcriptome data^42^.

The primary aim of the current study was to evaluate the suitability of *Nos2* ^−/−^ mice for TB drug development. Analysis of pathology over time revealed that lung lesions were homogeneously distributed and consistently developed central necrosis and hypoxia. Because the time window for onset of patient-like pathology is well defined between 42 and 56 days post-infection, we initiated drug administration at 42 days in the control group and at 56 days in the study group to specifically determine the impact of hypoxic necrotizing lesions on the outcome of monotherapy (Fig. 1a). A panel of nine drugs, either in clinical use or under clinical development was chosen for which efficacy data in humans or animal models are already available. The first-line TB drug INH showed low bactericidal activity in hypoxic necrotizing lesions (Fig. 3a). After an initial decline, the bacterial burden in lungs remained constant indicating the presence of drug-tolerant *M. tuberculosis* as validated by absence of genetic drug resistance after plating organ homogenates on agar supplemented with INH. It is tempting to speculate that the drug-tolerant subpopulation might be in a non-replicating state during which susceptibility to the cell wall inhibitor INH is lost. Evidence is increasing that bacterial persistence is a state actively maintained by a pool of intracellular stress responses allowing for prompt adaptation^43^. Whether INH-induced persistence of Mtb in *Nos2* ^−/−^ mice compares to classic non-replicating survival described in culture models^27, 28^ or indeed is actively maintained^44^ will be addressed in the context of our future work evaluating TB multi-drug regimens in the *Nos2* ^−/−^ model.

The rifamycins RIF and RPT showed strongest bactericidal activity among all drugs tested in our experiments. RPT was more potent prior to the onset of necrosis and hypoxia, whereas both drugs demonstrated comparable efficacy once patient-like pathology had established itself (Fig. 3b). Other murine models have suggested that RPT has roughly a 4-fold greater potency than RIF^15^. Our experiments indicate that hypoxic necrotizing lesions of *Nos2* ^−/−^ mice render RIF more potent but do not affect RPT efficacy (Fig. 3b). Intriguingly, RIF has been found to accumulate in the caseum of human TB lesions over time^21^. This could explain its increased efficacy in our experiments. In turn this would imply that RPT does not accumulate in lesions of *Nos2* ^−/−^ mice or in patients, which remains to be verified experimentally. Thus, performance of RIF and RPT in the *Nos2* ^−/−^ model closely mimics clinical outcome^8^.

Gradual oxygen depletion or nutrient starvation induces a state of non-replicating drug tolerance in *M. tuberculosis* ^27, 28^. Moreover, oxygen limitation renders *M. tuberculosis* susceptible to high concentrations of MTZ, which may not be achievable in humans at a sustainable level^28^. The drug was moderately active in late-stage TB lesions in *Nos2* ^−/−^ mice (Fig. 3c) indicating that a hypoxic non-replicating subpopulation resides in advanced caseous lesions. MTZ is active against *M. bovis* in rabbits^7^ and nonhuman primates^45^. Except for the late chronic stage of TB in BALB/c mice this drug lacks demonstrable activity in murine models including C3HeB/FeJ mice^13, 46^. MTZ may have led to earlier sputum smear and culture conversion in a clinical trial but was too neurotoxic for long-term use^47^. Two newer nitroimidazoles, PTM and DLM exhibit mycobactericidal activity both in growing and in hypoxic non-replicating bacilli and hence are more promising candidates against *M. tuberculosis* ^48^. DLM has been approved for patients with multidrug resistant TB and PTM is currently undergoing late stage clinical development. Both drugs expressed comparable activity in hypoxic necrotizing lesions of *Nos2* ^−/−^ mice (Fig. 3d,e) supporting their further evaluation.

The drug candidate, LZD, with demonstrable efficacy in extensively drug-resistant TB patients^38^, reduced the *M. tuberculosis* load in lungs of *Nos2* ^−/−^ mice prior to onset of necrosis and hypoxia and was bacteriostatic thereafter (Fig. 3f). Surprisingly this drug reduced occurrence of lung lesions including necrosis and completely abolished hypoxia (Fig. 4f). LZD has been shown to have immunomodulatory and anti-inflammatory effects^49, 50^, which may have supported pathologic alterations observed in our experiments. In C3HeB/FeJ mice a linear reduction of the bacterial burden in lungs over 8 weeks of LZD monotherapy was observed originally^13^. Using larger experimental groups, however, it was found that C3HeB/FeJ mice segregate into two groups of LZD responders either showing reduced c.f.u. in lungs similar to BALB/c mice or revealing a bacteriostatic profile^14^. Our results are comparable to the latter group of C3HeB/FeJ mice likely characterized by large caseous necrotic lesions^18^.

In general, MFX was strongly bactericidal in *Nos2* ^−/−^ mice, but roughly 10-fold less effective after onset of necrosis and hypoxia (Fig. 3g). Reduced activity was likely caused by insufficient lesion penetration of the drug as demonstrated in TB patients^21^. Several clinical trials to evaluate new TB drugs and treatment regimens have generated disappointing results^8–10^. The REMox TB trial, one of the largest clinical studies in the field, assessed whether replacement of INH or EMB in the standard regimen RIF/INH/EMB/PZA by the DNA-gyrase inhibitor MFX could shorten treatment time to 4 months. This trial was based on promising results from various murine models^51–53^ and on clinical data indicating a moderately higher rate of sputum conversion when EMB or INH were substituted by MFX^54–58^. Although initial murine studies failed to predict the clinical outcome of shorter MFX treatment time, carefully designed mouse experiments mimicked clinical results, underscoring the value of appropriately designed small rodent models for TB drug development^59^. TB drug testing in C3HeB/FeJ mice and new insights into drug penetration of TB lesions in patients and rabbits led to the conclusion that animal models showing patient-like TB pathology are key in overcoming the disconnect between pre-clinical and clinical studies^60^. In contrast to MFX, BTZ043 possessed consistent potency in *Nos2* ^−/−^ mice regardless of pulmonary pathology (Fig. 4h) suggesting uniform tissue distribution and sufficient lesion penetration. The profound bactericidal activity of BTZ043 in the *Nos2* ^−/−^ model, ranging between the most potent TB first-line drugs, INH and RIF, warrants further investigation of this and related benzothiazinones^61^.

In conclusion, unlike other murine models, *Nos2* ^−/−^ mice with patient-like pathology in principle correctly predicted clinical outcomes of RIF/RBT^8^, MTZ^47^, MXF^9, 10^, and potentially LZD^38^ (Supplementary Table S1). Results obtained for PTM, DLM and BTZ043 in *Nos2* ^−/−^ mice support development and consideration of these drugs for standard TB chemotherapy. Note that drugs interfering with host NO production or requiring activation by NO may not be suitable for testing in *Nos2* ^−/−^ mice due to their inability to produce NO in immune cells, which represents a limitation of this model. The development of new drugs and regimens for shortening the treatment of TB requires understanding the impact of specific drugs on specific types of lesions coexisting in patients. TB lesions in humans are very complex and often form a continuum of multiple discrete types of pathologies ranging from pneumonic infiltrates to dense consolidations to cavitation. No single model will suffice for understanding the impact of a new drug or regimen on all these types of pathologies. Yet, the *Nos2* ^−/−^ mouse with a patient-like pathology of lung granulomas and uniform disease progression provides a highly consistent and predictive model for this crucial type of pathology in TB drug development.

## Methods

### Bacterial strains and growth conditions

*M. tuberculosis* H37Rv (American Type Culture Collection, #27294) was grown in Middlebrook 7H9 broth (Becton Dickinson) supplemented with albumin-dextrose-catalase enrichment (Becton Dickinson), 0.2% glycerol, 0.05% Tween 80 or on Middlebrook 7H11 agar (Becton Dickinson) containing 10% v/v oleic acid-albumin-dextrose-catalase enrichment (Becton Dickinson) and 0.2% glycerol. Infection stocks were prepared from mid-log phase cultures. For c.f.u. determinations, serial dilutions were performed in PBS/0.05% Tween 80 and plated onto Middlebrook 7H11 agar. Plates were incubated at 37 °C for 3–4 weeks prior to c.f.u. counting.

### Animal experiments

Female C57BL/6 *Nos*2^−/−^ mice were bred in-house and maintained under specific pathogen-free conditions. Eight- to ten-week-old animals were anesthetized (ketamine 65 mg/kg, acepromazine 2 mg/kg, xylazine 11 mg/kg) and infected by injecting 1,000 c.f.u. of *M. tuberculosis* in 20 μl PBS into the ear dermis. At 14 and 21 days post-infection each mouse received 0.5 mg of monoclonal anti-tumour necrosis factor alpha antibody (purified from MP6-XT22 cultures) by i.p. injection. Two hours before euthanasia animals received 60 mg/kg pimonidazole hydrochloride i.p. to allow for detection of hypoxic regions in organ sections.

### Ethical statement

All animal studies have been ethically reviewed and approved by the State Office for Health and Social Services, Berlin, Germany. Experimental procedures were carried out in accordance with the European directive 2010/63/EU on Care, Welfare and Treatment of Animals.

### Drugs, formulations and administration

INH, LZD, RIF, RPT, MXF and MTZ were purchased from Sigma and formulated in 0.4% methylcellulose. Rifamycins were dissolved in dimethyl sulfoxide prior dilution in 0.4% methylcellulose. The final concentration of dimethyl sulfoxide did not exceed 5%. BTZ043^40^, PTM and DLM^62, 63^ were synthesized in-house and formulated in 0.25% carboxymethyl cellulose/0.05% Tween 80 (BTZ043) or in 10% hydroxypropyl-β-cyclodextrine/10% lecithin (PTM, DLM)^35, 64^. Drug formulations stored at 4 °C were administered in 0.2 ml per dose by oral gavage for 6 days per week.

### Staining procedures and histopathology

The left lung lobe of mice was fixed using 4% paraformaldehyde in PBS and embedded in paraffin. Organ sections (2–3 μm) were deparaffinised and subjected to haematoxylin and eosin (H&E) staining, Trichrome staining (Sigma), hypoxia staining (Hypoxyprobe-1 kit, Hypoxyprobe Inc.) or Ellis staining (Sigma) to visualize acid-fast bacilli in tissues; staining was performed according to the manufacturer’s instructions. Tissue sections were analysed using a Zeiss Axio Imager Z1 with CCD AxioCam. A researcher blinded to the study groups scored 16 individual stained sections of each organ in study groups of five mice per time point. Central necrosis of lesions was defined as a lighter pink region indicating tissue consolidation surrounded by granulomatous inflammatory infiltrate. Lesions were considered hypoxic when pimonidazole adducts in their central region could be visualized by a brown stain.

### Bacterial burden of mouse lungs and drug-resistant colonies

Mice were euthanized at dedicated time points and superior, middle inferior and post-caval lobes were removed and homogenized in 1 ml PBS/0.05% Tween 80. Serial dilutions of organ homogenates were plated onto Middlebrook 7H11 agar and in addition on agar supplemented with 0.4% activated charcoal for all time points during chemotherapy. Plates showing higher c.f.u. counts were used for data analysis. To determine the level of drug resistance at the last time point of each experiment, ¼ of the lung homogenate was plated onto Middlebrook 7H11 agar containing 4 μg/ml INH, 0.4 μg/ml RIF, 0.4 μg/ml RPT or 8 μg/ml LZD. Plates were examined after 4 weeks of incubation at 37 °C and kept until week 8 for re-examination. The limit of detection for drug-resistant colonies was 4 c.f.u. per organ.

## Electronic supplementary material

Supplementary Information
