# Supplementary material for: NOS2-deficient mice with hypoxic necrotizing lung lesions predict outcomes of tuberculosis chemotherapy in humans
Source: Sci Rep. 2017 Aug 18;7:8853. doi: 10.1038/s41598-017-09177-2 (PMC5562869; doi:10.1038/s41598-017-09177-2)
Supplement: Supplementary file 1 — Supplementary Information [file 41598_2017_9177_MOESM1_ESM.pdf]

Supplementary Information

NOS2-deficient mice with hypoxic necrotizing lung lesions predict outcomes of tuberculosis chemotherapy in humans

Martin Gengenbacher,* Maria A. Duque-Correa, Peggy Kaiser, Stefanie Schuerer, Doris Lazar, Ulrike Zedler, Stephen T. Reece, Amit Nayyar, Stewart T. Cole, Vadim Makarov, Clifton E. Barry III, Véronique Dartois, and Stefan H. E. Kaufmann*

*Correspondence: **Martin Gengenbacher**, Public Health Research Institute, Rutgers, The State University of New Jersey, 225 Warren Street, Newark, NJ 07103, USA, mg1435@njms.rutgers.edu; **Stefan H.E. Kaufmann**, Max Planck Institute for Infection Biology, Department of Immunology, Chariteplatz 1, 10117 Berlin, Germany, +49 30 28460 500, kaufmann@mpiib-berlin.mpg.de

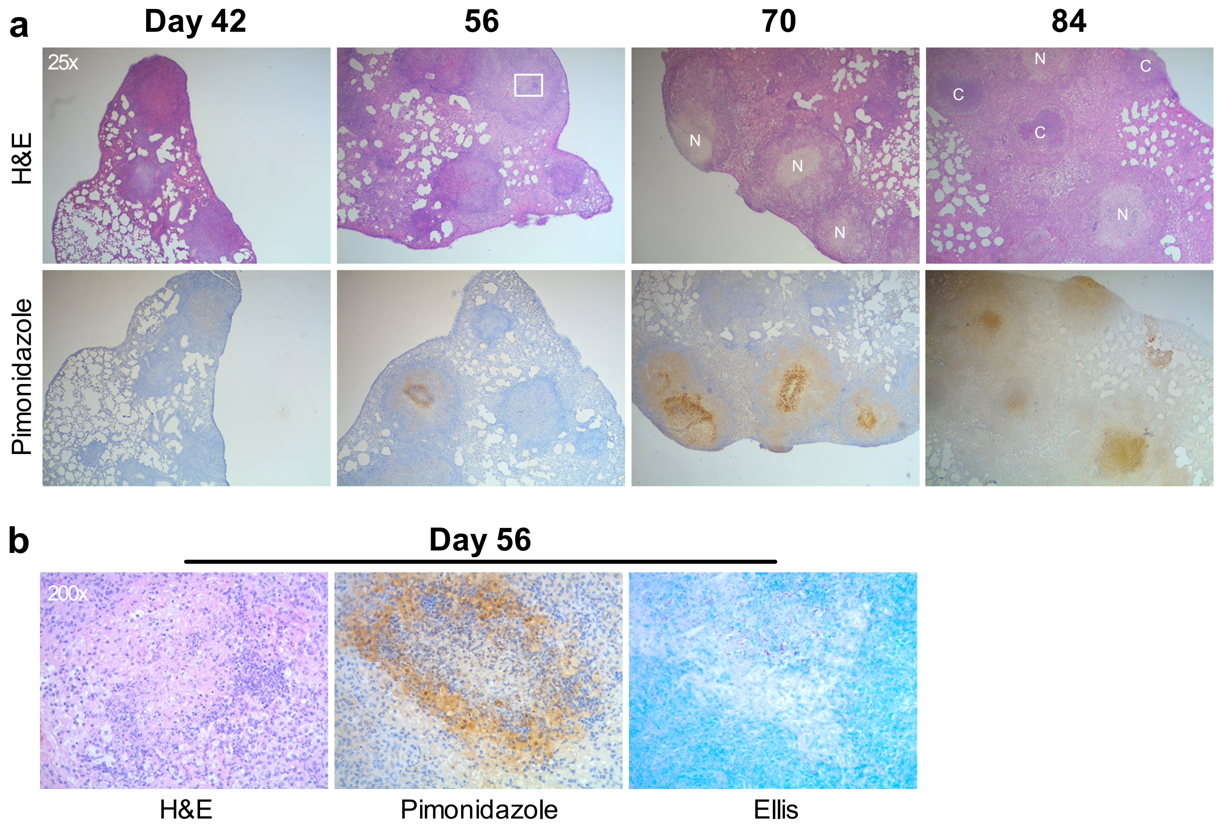

Supplementary Figure S1. Temporal development of pulmonary pathology in *Nos2*$^{-/-}$ mice with TB. a, Lung sections of *Nos2*$^{-/-}$ mice 42, 56, 70 and 84 days post-intradermal *M. tuberculosis* infection. H&E stained (upper panel) and corresponding consecutive section (lower panel) showing hypoxic regions (brown) as visualized pimonidazole adducts. Images were taken at 25-fold magnification. **b**, Lesion centre (**a**, white square) at 56 days showing onset of necrosis (left), hypoxic centre (middle panel, brown adducts) and presence of *M. tuberculosis* (right panel, violet rods visualized by Ellis staining). Images were taken at 200-fold magnification. C, caseation; N, necrosis; H&E, haematoxylin & eosin.

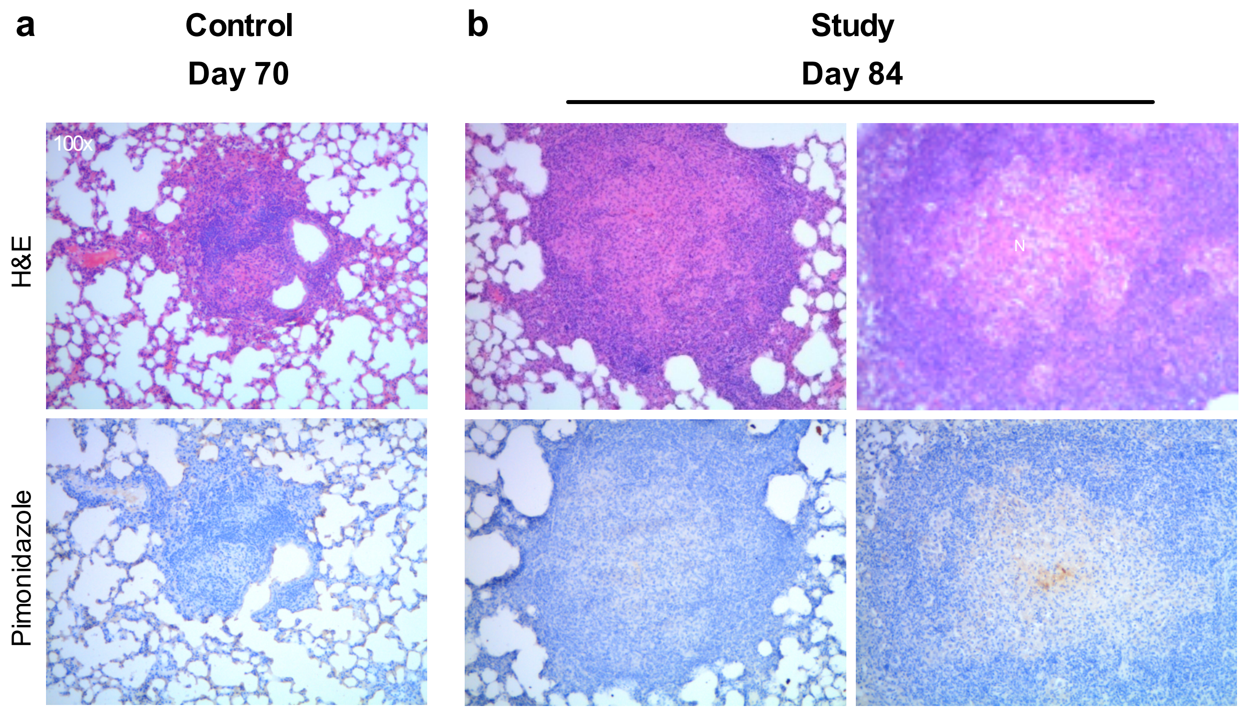

Supplementary Figure S2. Lung lesions of *M. tuberculosis*-infected *Nos2*$^{-/-}$ mice after linezolid monotherapy. a, Drug treatment was initiated before onset of human-like pathology (day 42). Lung sections of *Nos2*$^{-/-}$ mice after 4 weeks chemotherapy at 70 days post-intradermal *M. tuberculosis* infection. **b**, Drug treatment was initiated after onset of human-like pathology (day 56). Lung sections of *Nos2*$^{-/-}$ mice after 4 weeks chemotherapy at 84 days post-intradermal *M. tuberculosis* infection. Occasionally occurring necrotic lesion with minor presence of hypoxia is shown on the right. H&E stained (upper panel) and corresponding consecutive section (lower panel) showing hypoxic regions in brown as pimonidazole adducts. Images were taken at 100-fold magnification. N, necrosis; H&E, haematoxylin & eosin.

Supplementary Table S1. Efficacy of TB monotherapy in different mouse models and in humans.

	Mouse models					Humans
Drug	Dose mg/kg	BALB/c*	C3HeB/FeJ*	*Nos2*$^{-/-}$ Control*	*Nos2*$^{-/-}$ Study*	Early bactericidal activity $\log_{10}$ CFU/ml per day
INH[13,65]	25	-2.2	-1.4	-3.1	-1.9	0.67 (300 mg, days 0-2)<br>0.16 (300 mg, days 2-7)
RIF[15,66]	10	-2.0	-0.4	-2.3	-3.7	0.12 (300 mg, days 0-2)<br>0.10 (300 mg, days 2-5)
RIF[15,66]	20	-2.8	-2.0	-3.6	-4.5	0.22 (600 mg, days 0-2)<br>0.20 (600 mg, days 2-5)
RPT[15,66]	5	-1.9	-3.2	-4	-3.6	0.19 (300 mg, days 0-2)<br>0.17 (300 mg, days 2-5)
RPT[15,66]	10	-3.7	-3.3	-4.1	-4.2	0.24 (600 mg, days 0-2)<br>0.17 (600 mg, days 2-5)
MTZ[14]	400	-0.1‡	0.0‡	+1.4	+0.4	
PTM[35,67]	75	-1.9§		-1.1	-1.3	0.11 (200 mg, days 0-2)<br>0.11 (200 mg, days 2-14)
DLM[36,68]	1	-1.7¶		-1.7	-1.2	0.14 (200 mg, days 0-2)<br>0.04 (200 mg, days 2-14)
LZD[13,65]	100	-2.2	-1.1	-0.8	-0.3	0.18 (600 mg, days 0-2)<br>0.09 (600 mg, days 2-7)
MXF[39,69]	200	-5.0#		-3.3	-2.3	0.27 (400 mg, days 0-5)
BTZ043[40]	50	-0.8‖		-2.7	-2.5	

1.5 – 1	1 – 0.5	0.5 – -0.5	-0.5 – -1	-1 – -1.5	-1.5 – -2	-2 – -2.5	-2.5 – -3	-3 – -3.5	-3.5 – -4	< -4

*$\log_{10}$ reduction of lung CFU after 4 weeks of monotherapy relative to the bacterial burden prior treatment; ‡500 mg kg^{-1}; §6 weeks treatment, C57BL/6 mice, 100 mg kg^{-1}; ¶ICR mice; # High-dose systemic infection model, inbred Swiss-Webster mice, 100 mg kg^{-1}; ‖37.5 mg kg^{-1}, chronic model;

Supplementary references:

65 Dietze, R. *et al.* Early and extended early bactericidal activity of linezolid in pulmonary tuberculosis. *Am. J. Respir. Crit Care Med* **178**, 1180-1185 (2008).

66 Sirgel, F. A. *et al.* The early bactericidal activities of rifampin and rifapentine in pulmonary tuberculosis. *Am. J. Respir. Crit Care Med* **172**, 128-135 (2005).

67 Diacon, A. H. *et al.* Early bactericidal activity and pharmacokinetics of PA-824 in smear-positive tuberculosis patients. *Antimicrob. Agents Chemother* **54**, 3402-3407 (2010).

68 Diacon, A. H. *et al.* Early bactericidal activity of delamanid (OPC-67683) in smear-positive pulmonary tuberculosis patients. *Int. J. Tuberc. Lung Dis* **15**, 949-954 (2011).

69 Pletz, M. W. *et al.* Early bactericidal activity of moxifloxacin in treatment of pulmonary tuberculosis: a prospective, randomized study. *Antimicrob. Agents Chemother* **48**, 780-782 (2004).
